# Efficient regeneration and improved sonication-assisted *Agrobacterium* transformation (SAAT) method for *Catharanthus roseus*

**DOI:** 10.1007/s13205-016-0593-5

**Published:** 2017-04-11

**Authors:** Pravej Alam, Zainul Abdeen Khan, Malik Zainul Abdin, Jawaid A. Khan, Parvaiz Ahmad, Shereen F. Elkholy, Mahmoud A. Sharaf-Eldin

**Affiliations:** 1 Sara Alghonaim Research Chair (SRC), Biology Department, College of Science and Humanities, Prince Sattam bin Abdulaziz University (PSAU), Alkharj, 11942 Kingdom of Saudi Arabia; 2 Department of Biosciences, Faculty of Natural Sciences, Jamia Millia Islamia, New Delhi, 110025 India; 3 Department of Biotechnology, Faculty of Science, Centre for Transgenic Plant Development, Jamia Hamdard, New Delhi, 110062 India; 4 Department of Botany and Microbiology, Faculty of Science, King Saud University, Riyadh, 11451 Saudi Arabia; 5 Department of Botany, S.P. College, Srinagar, 190001 Jammu and Kashmir India; 6 Plant Transformation and Biopharmaceuticals Lab, Agricultural Genetic Engineering Research Institute (AGERI), Agricultural Research Centre (ARC), Giza, Egypt; 7 Department of Medicinal and Aromatic Plants Research, National Research Centre (NRC), Cairo, 12622 Egypt

**Keywords:** *Catharanthus roseus*, Internode, Hypocotyl, Regeneration, SAAT, BAP, NAA, *Agrobacterium*, GFP

## Abstract

*Catharanthus roseus* is an important medicinal plant known for its pharmacological qualities such as antimicrobial, anticancerous, antifeedant, antisterility, antidiabetic activities. More than 130 bioactive compounds like vinblastine, vindoline and vincristine have been synthesized in this plant. Extensive studies have been carried out for optimization regeneration and transformation protocols. Most of the protocol described are laborious and time-consuming. Due to sophisticated protocol of regeneration and genetic transformation, the production of these bioactive molecules is less and not feasible to be commercialized worldwide. Here we have optimized the efficient protocol for regeneration and transformation to minimize the time scale and enhance the transformation frequency through *Agrobacterium* and sonication-assisted transformation (SAAT) method. In this study, hypocotyl explants responded best for maximal production of transformed shoots. The callus percentage were recorded 52% with 1.0 mg L^−1^ (BAP) and 0.5 mg L^−1^ (NAA) while 80% shoot percentage obtained with 4.0 mg L^−1^ (BAP) and 0.05 mg L^−1^ (NAA). The microscopic studies revealed that the expression of GFP was clearly localized in leaf tissue of the *C. roseus* after transformation of pRepGFP0029 construct. Consequently, transformation efficiency was revealed on the basis of GFP localization. The transformation efficiency of SAAT method was 6.0% comparable to 3.5% as conventional method. Further, PCR analysis confirmed the integration of the *npt*II gene in the transformed plantlets of *C. roseus*.

## Introduction

*Catharanthus roseus* (L.) is a dicotyledonous medicinal plant naturalized in many countries like India, Saudi Arabia, South Africa, USA, Australia and most of the European region. The plant is cultivated worldwide for an ornamental purpose (Mujib et al. 2014). The important anticancer pharmaceuticals, vinblastine and vincristine, are also produced in very small quantities within the aerial parts of the *Catharanthus roseus*. The high cost of isolating the drugs has led to research efforts to increase the alkaloid content of *C. roseus* cell cultures, tissue cultures and seedlings.

The metabolic engineering of *C. roseus* strives is used to overcome the strict regulation of the alkaloid biosynthetic pathway. The alkaloid biosynthesis pathway in *C. roseus* is very complex and produces approximately 130 different alkaloids (Morgan et al. 2000). The high pharmaceutical cost associated with vinblastine and vincristine is a result of the very low quantities in which they are produced in planta and the isolation of these compounds from extracts. A total of 1000 kg of leaf and stem materials are required to produce 1 g and 20 mg vinblastine and vincrestine, respectively, (Facchini and De Luca 2008). Biosynthesis of these compounds occurs in five different subcellular compartments and three or more different cell types. It requires at least 35 intermediates and 30 enzymatic steps for biosynthesis of vinblastine and vincristine (Schröder et al. 1999; Lange et al. 2000; Morgan et al. 2000; Peebles et al. 2009). Due to low content (~0.0003%) of vinblastine and vincristine and expensive cost, the drugs could not be commercialized in global market. The straight chemical synthesis is not feasible due to chemical structure complexity and stereochemistry (Lange et al. 2000). Several investigators have carried out biotechnological efforts at in vitro level to elucidate the biosynthetic pathway and its genes to enhance the production of these drugs by genetic modulation of plant (van Der Heijden et al. 2004; Liscombe et al. 2010). Due to ineffective transformation and regeneration protocols, the rate of transformation is quite low. Therefore, we have developed an efficient regeneration and sonication-assisted *Agrobacterium tumefaciens*-mediated transformation method for *C. roseus*.

## Materials and methods

### Plant material and seed germination

Seeds of *Catharanthus roseus* were procured from Herbal Garden, Jamia Hamdard, New Delhi, India. The seeds were sterilized with mercuric chloride (0.1%) for 2 min, followed by washing with double-distilled sterile water. Thereafter, sterilized seeds were cultured on MS basal medium containing sucrose (3.0% w/v) and agar (0.8% w/v) for germination at pH 5.8 and autoclaved for 1 bar pressure at 20 min. The seedlings of *C. roseus* were maintained at 25 ± 2 °C under a 16 h (hour) light and 8 h dark regimes.

### Optimization of regeneration protocol for *Catharanthus roseus*

Small young in vitro raised plantlets were collected from 10 days old healthy *Catharanthus roseus* plants, cut into 1–1.5 cm hypocotyl and 3–5 mm internode long in size. MS medium (Murashige and Skoog 1962) was supplemented with various plant growth regulators. NAA (naphthalene acetic acid) and BAP (6-benzylaminopurin) in combination with different concentration have been used for callus and shoot induction (Tables 1, 2). There were 17 treatments of BAP and NAA used for callus induction for the explants; casein hydrolysate (150 mg L^−1^), sucrose (3%), proline (250 mg L^−1^) and agar 0.8% were inoculated in callus induction medium. The cultures were incubated under fluorescent lights with 2300 l× for 16 h at a temperature of 25 ± 2 °C.

**Table 1:** Effect of different concentrations of BAP sand NAA on % callus induction in the hypocotyl explants of *C. roseus*

Growth regulator (mg L^−1^)		Callus formation (%)
BAP	NAA	
0.0	–	–
0.1	–	–
0.5	–	–
1.0	–	–
–	0.0	–
–	0.1	1.00 ± 0.35
–	0.5	1.50 ± 0.57
–	1.0	2.0 ± 0.43
0.1	0.5	6.66 ± 1.55
0.1	1.0	10.66 ± 1.52
0.1	1.5	3.00 ± 0.51
0.5	0.5	4.33 ± 0.58
0.5	1.0	23.00 ± 2.35
0.5	1.5	25.66 ± 0.57
1.0	0.5	52.00 ± 2.00
1.0	1.0	32.66 ± 2.08
1.0	1.5	24.66 ± 2.51

Each value is the mean ± standard error (*n* = 5)

**Table 2:** Effect of various concentrations of BAP and NAA on the shoot induction from hypocotyls explant of *C. roseus*

Treatments	BAP (mg L^−1^)	NAA (mg L^−1^)	Shoot induction (%)	No. of shoots per explants
MS0	–	–	–	–
MS1	1.0	–	–	–
MS2	1.5	–	–	–
MS3	2.5	–	–	–
MS4	2.0	–	–	–
MS5	3.0			–
MS6	4.0			–
MS7	–	0.01	–	–
MS8	–	0.02	–	–
MS9	–	0.03	–	–
MS10	–	0.04	–	–
MS11	–	0.05	–	–
MS12	1.0	0.01	–	–
MS13	1.0	0.02	–	–
MS14	1.0	0.03	–	–
MS15	1.0	0.04	–	–
MS16	1.0	0.05	–	–
MS17	2.0	0.01	–	–
MS18	2.0	0.02	–	–
MS19	2.0	0.03	–	–
MS20	2.0	0.04	–	–
MS21	2.0	0.05	–	–
MS22	3.0	0.01	–	–
MS23	3.0	0.02	+	–
MS24	3.0	0.03	+	–
MS25	3.0	0.04	++	–
MS26	3.0	0.05	++	–
MS27	4.0	0.1	++	–
MS28	4.0	0.02	45	12 ± 0.55
MS29	4.0	0.03	53	19 ± 2.15
MS30	4.0	0.04	65	25 ± 2.15
MS31	4.0	0.05	80	35 ± 0.70
MS32	5.0	0.1	40	15 ± 0.37
MS33	5.0	0.02	35	12 ± 0.52
MS34	5.0	0.03	20	10 ± 0.40
MS35	5.0	0.04	15	9.0 ± 0.55
MS36	5.0	0.05	12	6.0 ± 0.65

Each value is the mean ± standard error (*n* = 5)

In callus induction both hypocotyl and internode segments were used, but only hypocotyl induced the better callus growth while the internode generated moderate type of callus. After 10 days of incubation, the calli of hypocotyl explants were further cultured on shoot induction medium. We have optimized the 36 treatments for shoot induction medium (SIM) using the various concentration and combinations of naphthalene acetic acid (NAA) and 6-benzylaminopurin (BAP). After 15 days of culture on SIM and optimization of the best shoot induction frequency, the clusters of shoots were excised from the base of the callus and cultured on shoot elongation medium (MS basal). After two weeks, the apical dominance was observed in shoots with many plantlets. The shoots were thereafter cultured on rooting media with varying concentration of NAA (0.1–1.0 mg L^−1^) and IBA (0.1–1.0 mg L^−1^) in MS medium having sucrose (3% w/v), agar (0.8% w/v), proline (250 mg L^−1^) and casein hydrolysate (150 mg L^−1^).

### Determinition of optimal concentration of kanamycin for *C. roseus* transformants

To examine the transformed shoots from hypocotyl explants on shoot induction medium, a selection agent (antibiotics) was needed to check the false and putative transformants, hypocotyl explants were cultured on MS medium, containing various concentrations of kanamycin (5, 10, 15, 20, 30, 40, 50 60, 70, 80, 100 mg L^−1^). The calli were induced only in those have transformed and adventitious shoots thereafter were counted with respect to transformation frequencies on shoot induction medium.

### Transformation of *C. roseus* hypocotyl explants

#### Conventional and sonication-assisted *Agrobacterium*-mediated transformation (SAAT)

**Fig. 1:**
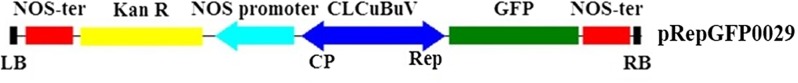
Schematic representation of the expression vector (pGreen0029) containing green fluorescent protein tagged with CLCuBuV promoter. *CP* coat protein, *Rep* replication initiation protein, *CLCuBuV* cotton leaf curl Burewala virus, *LB* left-border sequence of T-DNA, *RB* right-border sequence of T-DNA, *GFP* green fluorescent protein, *Kan R* Kanamycin resistance gene

*Agrobacterium* strain LBA4404 harbouring GFP gene construct cloned into pGreen0029 (pRepGFP0029) tagged with newly identified strong constitutive promoter (Fig. 1, Khan et al. 2015) was obtained from Plant virology laboratory, Department of Biosciences, Jamia Millia Islamia, New Delhi, India. *Agrobacterium tumefaciens* strain LBA4404 harbouring pRepGFP0029 along with pSoup helper plasmid, culture in 50 mL of YEB (yeast extract broth) medium with Kan (kanamycin, 50 mg L^−1^) and Rif (rifampicin 10 mg L^−1^) at 27 °C until an OD_600_ nm reached 0.8 following the method of Khan et al. (2015). Overnight culture was centrifuged and pellet was re-suspended in 100 mL MS liquid with 3% sucrose (w/v). For conventional method, hypocotyl explants were thereafter immersed in *Agrobacterium tumefaciens* LBA4404 culture (OD_600_ 0.8) for 1 h at normal room temperature with a slight shaking. After 1 h of *Agrobacterium* infection, the hypocotyl explants were evoked from medium and blot off on sterile tissue paper. Further, infected explants were cultured on co-culture medium [MS salt, sucrose (3%), proline (250 mg L^−1^), casein hydolysate (150 mg L^−1^) and agar (0.8%)]. at 28 °C in the dark for 4 days.

Similarly, overnight *Agrobacterium* culture previously mixed in MS liquid medium was again treated with hypocotyl explants under centre of bath in ultra-sonication unit (ImecoUltrasonics, India) for 10 min in microcentrifuge tubes. Hypocotyl explants of *C. roseus* were removed from micro centrifuge tubes, placed on sterile tissue paper to blot off the excess bacteria on the surface of the explants and cultured on same co-cultivation medium [MS salt, sucrose (3%), Proline (250 mg L^−1^), casein hydolysate (150 mg L^−1^) and agar (0.8%)] for 4 days. After 4 days of co-cultivation, hypocotyl explants were washed with double-distilled sterile water and blot dried with sterile tissue paper. After proper washing with sterile water, hypocotyl explants were further washed with cefotaxime (500 mg L^−1^) for 10 min to kill the excess growth of bacteria. This step was repeated twice and finally explants were washed with sterile water. Although the regeneration of the shoot was not induced directly, the explants were first cultured on pre-standardized callus and shoot induction medium in a sequential manner (Tables 1, 2). After proper attaining of cultured shoots on shoot induction selection medium (SISM), the induced shoots were cultured in pre-optimized root induction medium with selection agent (Tables 1, 2). To facilitate the optimization of the method of conventional and SAAT-based transformation methods of hypocotyl of *C. roseus*, several factors like pre-culture of explants, *Agrobacterium* density, infection time and co-cultivation period have been studied for achieving a high rate of transformants. The transformation frequency was counted on the basis of GFP localization and *npt*II gene integration of transformants grown on SISM using confocal microscopy and PCR assays.

The transformation efficiency was calculated according to the following formula:

$$ {\text{Transformation efficiency }}\left( \% \right) = \frac{\text{Number of GFP positive plants}}{{{\text{Number of explants inoculated with }}Agrobacterium}} \times 100. $$

### GFP Localization and PCR analysis of transformed leaves of *C. roseus*

The localization of green fluorescent protein (GFP) in the transformed leaves of *C. roseus* was carried out using inverted florescent microscope (Motic) and confocal laser scanning microscope (40.0X1.15OIL, Leica Microsystems, Germany). To observe the GFP in transformed leaves of *C. roseu*s, the AOTF (Acousto-Optical Tunable Filter) of 488 nm (at 40%) was used and the fluorescence emission collected between 501 and 598 nm as described earlier by Khan et al. (2015).

Genomic DNA from transformed leaves of *C. roseus* was extracted by employing the method of Doyle and Doyle (1990). The presence of *npt*II transgene signals in plant tissue was confirmed by PCR amplification using the specific primers set (forward 5′ATGCCCGATCGAGCTCAAGT3′ and reverse 5′TCGTCTGGCTGACTTTCGTCATAA3′). The PCR reaction mixture was carried out in 100 μL eppendorf tubes in Gstorm thermal cycler (Germany). The reaction mixture of total volume 25 μL contained 2X Taq polymerase assay buffer, 2.5 mM dNTP, 100 ng forward and reverse primers, 3U*Taq* DNA polymerase and template DNA (100 ng) with the following parameters: initial denaturation 94 °C, 5 min.; followed by 35 cycles; denaturation 94 °C, 1.0 min.; annealing 55 °C, 1.0 min; elongation 72 °C, 2.0 min with final extension 72 °C, 5 min.. PCR products were resolved on 1.0% agarose gel stained with ethidium bromide and visualized in UVI gel documentation system.

### Statistical analyses

All experiments were directed on the basis of three replicates using one-way analysis of variance (ANOVA) followed by DMRT (Duncan’s multiple range) by using the SPSS statistical software. The values are mean ± SE for three samples in each group were studied. *P* values at ≤0.05 were measured as significant.

## Results and discussion

### Establishment of in vitro regeneration for *C. roseus*

The seedlings of *C. roseus* raised aseptically from seeds were used as a source of explants. The hypocotyl (7 days) and internode (20 days) explants were designated and cultured on MS (Murashige and Skoog 1962) medium augmented with different plant growth regulators like naphthalene acetic acid (NAA) and 6-benzyl amino purine (BAP), either alone or in combinations of callus and shoot induction media (Tables 1, 2). The cultures were maintained at white fluorescent light (2300 l×) for 16 h with 25 ± 2 °C.

### Effect of BAP and NAA on callus induction

In order to study the effect of BAP and NAA on induction and growth of callus from the cultured hypocotyl and internodal explants of *C. roseus*, different concentrationsof BAP and NAA, along with proline (250 mg L^−1^) and casein hydrolysate (150 mg L^−1^) were tested singly as well as in combinations.

The callus induction responses of the hypocotyl explants ranged from 1.00 ± 0.35 to 52.00 ± 2.00 with the ranging concentration of BAP and NAA. But the internode explants showed moderate response on callus development with different combinations and concentration of BAP and NAA (Table 1). Single BAP or NAA treatments did not exhibit any significant response, as only small callus was initiated within 4 weeks of inoculation (Table 1). However, the treatment of cytokinins and auxins at various concentrations and combinations exhibited significant response in terms of callus induction, but with varying degree of callus growth. Among the various combinations tested, the medium comprised of MS with 1.0 mg L^−1^ BAP and 0.5 mg L^−1^ NAA proved to be the best with 52.00 ± 2.00% callus formation with hypocotyl explants (Table 1). On this medium, callus initiation was started from the edge of the explants after 4 days of inoculation. The responses of calli initiation and growth were decreased as the concentration of both BAP and NAA was dropped.

### Multiple shoot induction, elongation and rooting

The hypocotyl explants were further cultured on shoot induction medium (SIM) containing various combinations of BAP and NAA along with proline (250 mg L^−1^), casein hydrolysate (150 mg L^−1^) and sucrose (3%; MS basal medium contains BAP or NAA alone did not respond to morphogenesis (Table 2), whereas on shoot induction medium comprised of MS with various concentrations of BAP and NAA, they exhibited shoot induction on some selective concentration (Table 2). The highest concentration of BAP with a very low concentration of NAA was found to be quite beneficial for shoot induction. The MS medium with BAP (4.0 mg L^−1^) and NAA (0.05 mg L^−1^) evoked maximum response 35.0 ± 0.7 (80%) shoots per explant after 5 weeks of culture period. The shoot initiation was started after 15 days of callus culture. On lowering or increasing the concentration of either of the PGRs, the shoot regeneration frequency was declined (Table 2).

**Fig. 2:**
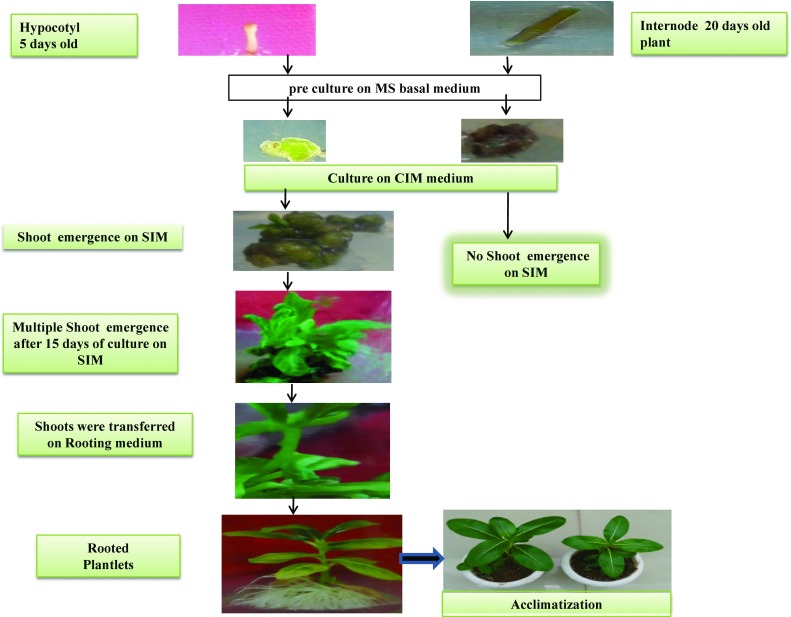
Stages of regenertion of *C. roseus* though internodal and hypocotyl segments

As the regenerated shoots on the optimal medium for shoot induction (MS, 4.0 mg L^−1^ BAP, 0.05 mg L^−1^ NAA, along with 250 mg L^−1^ proline, 150 mg L^−1^ casein hydrolysate and 3% sucrose) could not attain a suitable length to be utilized for rooting, the clusters of developed shoots were excised and cultured on growth regulator-free MS medium [MS basal along with proline (250 mg L^−1^), casein hydrolysate (150 mg L^−1^) and sucrose (3%)]. On this medium, elongation in the shoots accelerated after 2 weeks of culture, attaining a suitable length of 1.5 cm within 4 weeks (Fig. 2). The microshoots of *C. roseus* (size 2.0 cm) were excised from the shoot clusters and cultured on rooting medium to get complete plantlets.

The rooting medium was comprised of MS basal medium, with various concentrations of NAA or IBA (0.1, 0.5 and 1.0 mg L^−1^) along with casein hydolysate (150 mg L^−1^) and proline (250 mg L^−1^). IBA proved to be the best at 1.0 mg L^−1^ concentration and recorded 30.0 ± 0.36 roots per shoot were produced in 100% cultures in 5 weeks of culture period. On this rooting medium [MS + IBA (1.0 mg L^−1^)], the root induction was observed after 10 days of planting. While in NAA-supplemented medium, higher concentration of the growth regulator (1.0 mg L^−1^) has induced only 16.5 ± 0.23 roots/shoot (Table 3).

**Table 3:** Effect of growth regulators (NAA and IBA) on rooting of in vitro regenerated shoots of *C. roseus*

Growth regulators	Concentration (mg/L)	No. of roots/plantlet
IBA	0.1	5.0 ± 0.30
	0.5	12.3 ± 0.25
	1.0	30.0 ± 0.36
NAA	0.1	4.0 ± 0.41
	0.5	10.5 ± 0.52
	1.0	16.5 ± 0.23

Each value is the mean ± standard error (*n* = 5)

The morphogenic comebacks of micropropagated plant are mainly affected by the media component (s) and plant growth regulator concentration. In order to optimize the suitable regeneration frequency of *C. roseus* and to increase the possibility of rate of transformants, we therefore, optimized 17 and 36 media treatment (Tables 1, 4) for callus and shoot to check the regeneration ability of the hypocotyl and internodal explants of *C. roseus*. Hundred explants were inoculated on each treatment of MS medium with variousconcentration (s) of BAP and NAA (Tables 1, 4). Only hypocotyl explants were responding for good callus induction while for intermodal segment poorly developed calli were obtained (Fig. 2). On the basis of growth of calli, we have further cultured the calli of hypocotyl on shoot induction medium (SIM). The inducing of a proliferated calli of the explants showed profound changes in the developmental stages of the tissue and hence resulted in alteration in the basic architecture of cell and tissues for quiescent or fully differentiated cells. One of the most influencing factors in multiple shoot induction of hypocotyl explants is the modulation of endogenous auxin to cytokinin balance. Morphogenic responses thus exhibited in the form of shoots or roots correlated with the specific auxin and cytokinin ratio (Bennici et al. 1988; Thorpe 2006; Chawla and Wenzel 1987). In the present study, we have found that BAP at 4.0 mg L^−1^ and NAA 0.05 mg L^−1^ were considered the optimal concentrations to obtain maximum shoot for the hypocotyl explants of *C. roseus*. Increasing BAP and NAA concentrations beyond optimal level have shown a negative effect and the exhibited shoot get stunted in nature with the reduction in number of shoots (Table 2). These findings are in consonance with the results obtained earlier in *Erigeron breviscapus* (Liu et al. 2008), *Dioscorea nipponica* (Chen et al. 2007), *Sida cordifolia* (Sivanesan and Jeong 2007), *Aloysia polystachya* (Burdyn et al. 2006), *Pogostemon heyneanus* (Hembrom et al. 2006) and *Psoralea corylifolia* (Faisal and Anis 2006). Based on the findings, it could be stated that the BAP and NAA ensure in vitro regeneration of shoots from hypocotyl explants and synergism of BAP and NAA in appropriate concentration is promising for *C. roseus* micropropagation.

**Table 4:** Selection of number of transformants on shoot induction selection medium (SISM) containing kanamycin (Kan^r^) and GFP positive shoots after co-cultivation of *C. roseus* hypocotyl explants infected with *Agrobacterium tumefaciens* LBA4404

No. of explants	Kan^r^		GFP	
	SAAT	Conventional	SAAT	Conventional
50	4 ± 0.43	2 ± 0.33	3 ± 0.44	1 ± 0.32
50	3 ± 0.21	2 ± 0.3	3 ± 0.44	2 ± 0.32
50	4 ± 0.44	3 ± 0.18	4 ± 0.4	3 ± 0.31
50	3 ± 0.22	1 ± 0.22	2 ± 0.11	1 ± 0.32

Each value is the mean ± standard error (*n* = 3)

### Genetic transformation of *C. roseus*

The *Agrobacterium-*mediated transformation depends upon several parameters for generating the transformed lines of *C. rosues*. In this method, the lethal dose of kanamycin required for effective selection of transformed hypocotyl explants was determined by the culturing explants on MS basal medium containing various concentrations of kanamycin (0–100 mg L^−1^). Hypocotyl explants were cultured on shoot induction medium (SIM) for 4–5 days before they were transferred to the same medium containing various concentrations of kanamycin (SISM). There was an enormous growth of callus induced in control hypocotyl explants on the medium without kanamycin. No callus or shoot responded on the MS medium containing 40 mg L^−1^or higher concentration of kanamycin. Therefore, selection of kanamycin at 40 mg L^−1^was used after co-cultivation and maintained till the callus and shoots were obtained. Kanamycin at 40 mg L^−1^ resulted in the death of more than 80% hypocotyl explants; therefore, this concentration was selected as an optimal concentration for selection of transformed lines (data not shown).

### Transformation rate increment through pre-culture and co-cultivation

**Fig. 3:**
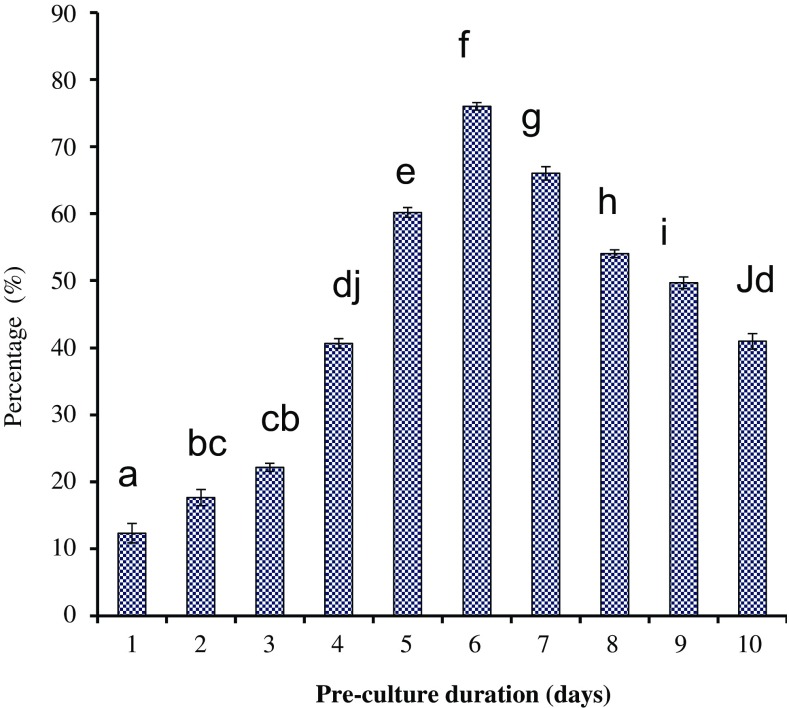
Influence of pre-culture duration on rate of transformation in *C. roseus*

To determine the high rate of transformation frequency, explants were pre-cultured for 1–7 days and the numbers of transformed explants were recorded. Pre-culture of explants in tolerable situations prior to *Agrobacterium* infection may improve the rate of transformation with increasing the cells number and competent for transgene insertion (Husaini 2009; Alam et al. 2014) Several investigations used pre-culture period of 3–10 days for different kinds of explants to adjust the rate of transformation in the regeneration media before *Agrobacterium* infection (Husaini 2009). Pre-culturing explants for 6 days on MS basal medium with proline (250 mg L^−1^) and casein hydolysate (150 mg L^−1^) was a prerequisite for the efficient transformation (76%) and helps in regeneration of explants with *Agrobacterium tumefaciens*. The use of proline (250 mg L^−1^) and casein hydolysate (150 mg L^−1^) in pre-culture medium functions as osmoprotectant and absorbent of extra salt and saves the explants from death during transformation and regeneration. Those explants which were not treated with pre-culture showed less transformation efficiency (20) on 6 days. In contrast, pre-cultured explants showed improved transformation rates to 76% (Fig. 3). It was due to the expansion of cell size in term of cell elongation and cell division which was more able to withstand bacterial treatment (Kiani et al. 2014).

Moreover, due to growth, the selectively dividing cells can be more easily transformed. Hypocotyl explants that had not been pre-cultured remained small in size (about 1.0 cm^2^ compared to 1.5 cm^2^ for pre-cultured explants) and were severely affected by bacterial treatment.

### Effect of co-cultivation duration on transformation

**Fig. 4:**
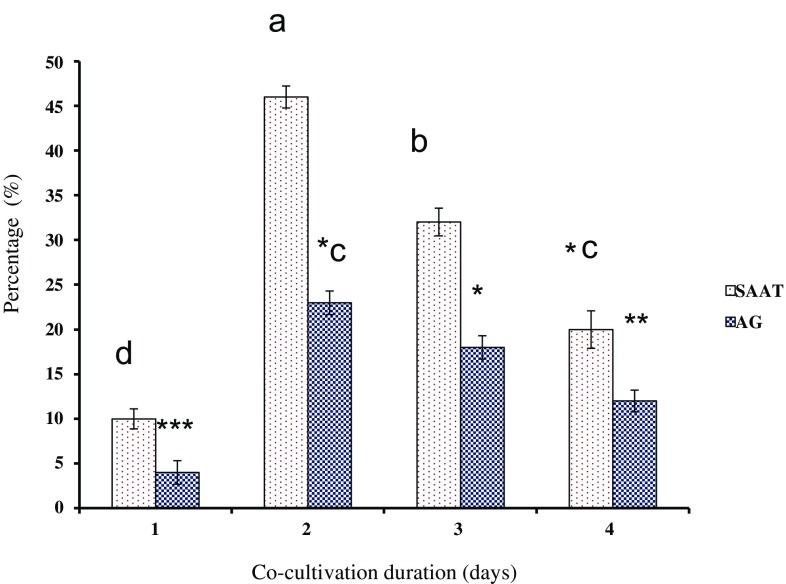
Effect of co-cultivation period on transformation rate in *C. roseus*

The transformation experiment was carried out to check the efficacy of *Agrobacterium* density, infection time and co-cultivation time period. In this study, we have randomly selected 30-min infection time of *Agrobacterium* and inoculum density of 0.6 OD_600_ nm of LBA4404 for *C. roseus*. Previous studies have shown that co-cultivation of explants was essential factor to achieve the maximal transformation rates with *Agrobacterium tumefaciens* in different plant species (Alam and Abdin 2011; Husaini 2009; Alam et al. 2014). On the basis of previous studies, therefore, we augmented co-cultivation period of hypocotyls explants with *A. tumefaciens* for 1, 2, 3 and 4 days (d). Furthermore, the co-cultivation period was assayed in the 2 days in dark at 27 °C in co-cultivation medium containing MS basal with proline (250 mg L^−1^) and casein hydolysate (150 mg L^−1^) which leads to higher rates of transformation as compared to the number of days of co-cultivation (Fig. 4). If the optical density of bacterial culture is more than 0.8 and co-cultivation period ≥3–4 days, the explants had more overgrowth of bacteria on the surface and leaching had been started. Throughout co-cultivation period, it is noted that an excessive number of bacterial growth caused stress on explants cells and inhibits the regeneration potential. Similarly, the lower number of bacteria (OD_600_ ≤ 0.8) minimized the rate of recurrence of Transfer DNA in plant cells (Montoro et al. 2003). In this study, 6-day-old hypocotyl explants produced sophisticated number of transformed shoots through callus culture. By increasing the bacterial optical density and time of infection (beyond 30 min in conventional and 10 min in SAAT), explants showed blackening and were dead after some time of co-cultivation, due to excessive growth of bacteria. Increasing the co-cultivation duration may enhance transformation events, but bacteria incline to colonize the explant topology triggering tissue necrosis and finally death of explants (Folta et al. 2006). In contrast to previous studies, we observed that *C. roseus* did not show significant differences in the transformation rates with co-cultivation for 1, 2, 3 days (Wang et al. 2012). In the present study, *Agrobacterium* infection was cultured for 30 min in MS liquid medium followed by co-cultivation for 48 h enhance the transformation rate 46% in SAAT as compared to 23% in conventional method (Fig. 4).

Transformed hypocotyl explants obtained from 6-day-old *C. roseus* seedlings were cultured in callus induction medium [MS salts, BAP (1.0 mg L^−1^), NAA (0.5 mg L^−1^) proline (250 mg L^−1^), casein hydrolysate (150 mg L^−1^) sucrose (3% w/v) and agar (0.8% w/v)]. After induction of calli, it was transferred to shoot induction medium (SIM) with concentration of 40 mg L^−1^. No callus growth or shoot regeneration was, however, observed on the medium with either 40 mg L^−1^ or higher concentrations of kanamycin in un-transformed explants. Transformed calli were further transferred to SISM containing MS salts, BAP (4.0 mg L^−1^), NAA (0.05 mg L^−1^) proline (250 mg L^−1^), casein hydrolysate (150 mg L^−1^), kanamycin (40 mg L^−1^), sucrose (3% w/v) and agar (0.8% w/v) to observe the selection pressure for induction of putative transformants. After one week of callus development, most of the un-transformed calli did not produce shoots and died, but some of the transformed calli induced shoots on SISM (Fig. 2). Out of 200 explants used for transformation experiments, only 14 kanamycin-resistant shoots were induced (Table 4). This finding could be due to plant genotype, pre-culture time, co-cultivation condition, bacterial density and use of osmoprotectant and absorbent.

### GFP localization and molecular analyses of *C. roseus* plantlets

**Fig. 5:**
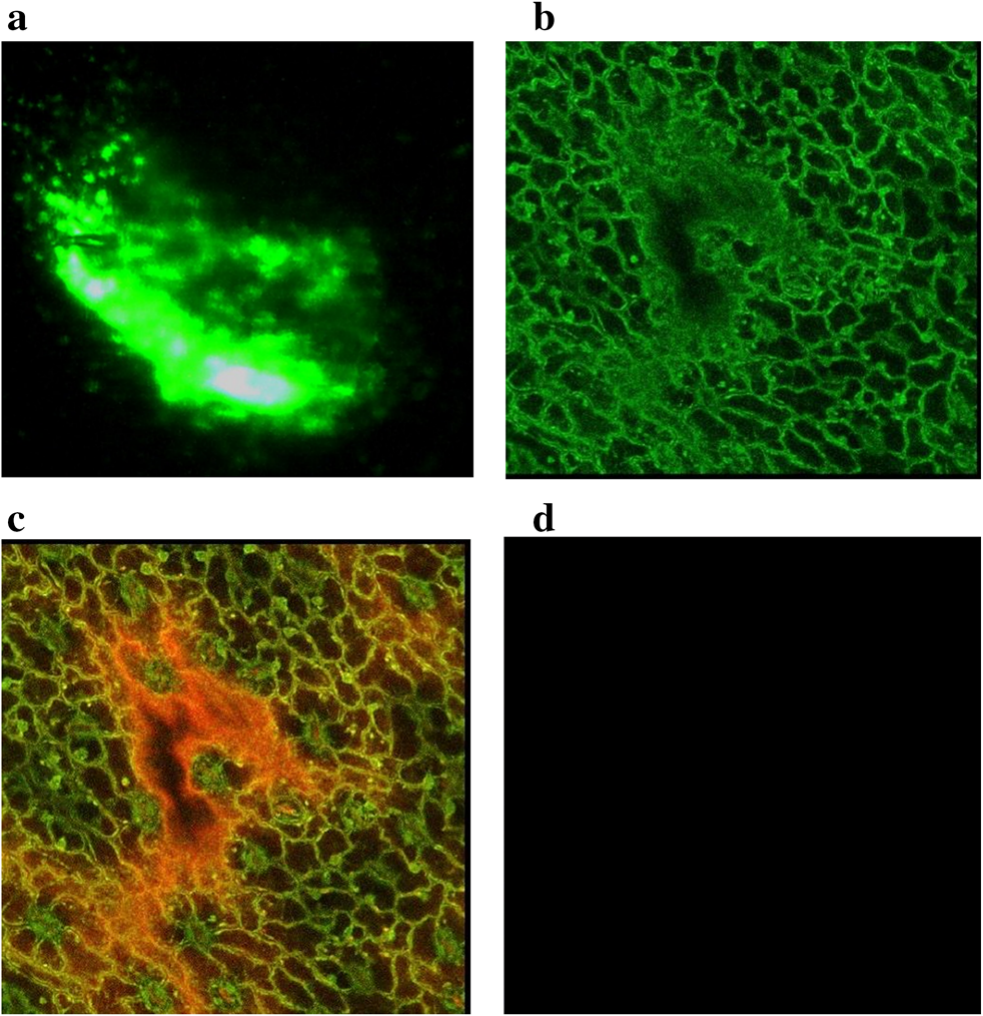
Visualization of green fluorescent protein in transformed *Catharanthus roseus* leaf.** a** Fluorescent microscope view,** b** confocal microscopic view,** c** GFP expression fused with chlorophyll (*red*),** d** un-transformed leaf

In the recent past, it was significant to know the transgene localization and level of expression in different cells, GFP based study achieved this task easily in short period of time after transfection of explants have been used. Therefore, we used GFP-based construct to optimize the rate of transformation frequency and analyse the localization of GFP at the cellular level in *C. roseus* using confocal laser scanning microscopy (CLSM) and florescent microscopy. The results revealed by confocal microscopy showed distinct subcellular localization of GFP expressing in cells of *C. roseus* leaves after *Agrobacterium*-mediated transformation (Fig. 5). Out of 14 kanamycin-resistant plantlets were tested for GFP localization, 12 plants gave the positive signals of GFP expression in SAAT while 7 were obtained using conventional method. The differences between SAAT and conventional method may due to the use of sonication process to integrate the construct in *C. roseus* genome (Table 4).

**Fig. 6:**
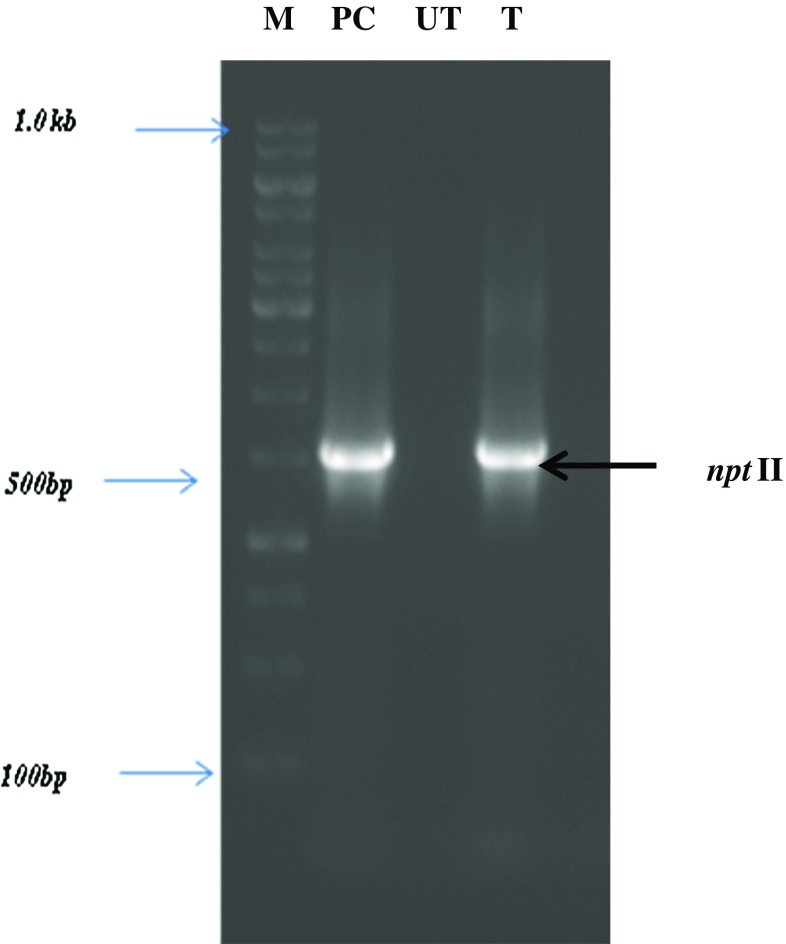
PCR analysis for the presence of the *npt*II gene in kanamycin-resistant *C. roseus* transgenic lines. Lanes: M: 1 kb ladder, PC-positive control of *npt*II from pRepGFP0029, UT-non-transgenic control plant, T-PCR positive Transformed *C. roseus* line

Genomic DNA from GFP positive and un-transformed leaves of *C. roseus* was extracted and PCR analysis was carried out using gene-specific primers for *npt*II. All the GFP positive transformants showed the presence of 500 bps *npt*II gene as a selectable marker gene (Fig. 6). The transformation efficiency was, thus, 6.0% obtained in SAAT than 3.5% in the conventional method. Our results were also consonant with the earlier study of Wang et al. (2012). The success of high rate in production of stable transformed shoots from hypocotyl explants reported in this study is obtained by influencing some parameters like explants types, bacterial strain and co-cultivation condition.
